# Microbial biofilm correlates with an increased antibiotic tolerance and poor therapeutic outcome in infective endocarditis

**DOI:** 10.1186/s12866-019-1596-2

**Published:** 2019-10-21

**Authors:** Enea Gino Di Domenico, Sara Giordana Rimoldi, Ilaria Cavallo, Giovanna D’Agosto, Elisabetta Trento, Giovanni Cagnoni, Alessandro Palazzin, Cristina Pagani, Francesca Romeri, Elena De Vecchi, Monica Schiavini, Daniela Secchi, Carlo Antona, Giuliano Rizzardini, Rita Barbara Dichirico, Luigi Toma, Daniela Kovacs, Giorgia Cardinali, Maria Teresa Gallo, Maria Rita Gismondo, Fabrizio Ensoli

**Affiliations:** 1 Clinical Pathology and Microbiology, San Gallicano Dermatology Institute, IRCCS, Istituti Fisioterapici Ospitalieri (IFO), via Elio Chianesi, 53 00144 Rome, Italy; 2 Laboratorio di Microbiologia Clinica, Virologia e Diagnostica delle Bioemergenze, Azienda Socio Sanitaria Territoriale Fatebenefratelli-Sacco, Polo Universitario, Via G.B. Grassi 74, 20157 Milan, Italy; 3 UOC Cardiochirurgia, Azienda Socio Sanitaria Territoriale Fatebenefratelli-Sacco, Polo Universitario, Via G.B. Grassi 74, 20157 Milan, Italy; 4 Laboratory of Clinical Chemistry and Microbiology, IRCCS Orthopedic Institute Galeazzi, Via R. Galeazzi 4, 20161 Milan, Italy; 5 Dipartimento di Malattie Infettive, Azienda Socio Sanitaria Territoriale Fatebenefratelli-Sacco, Polo Universitario, Via G.B. Grassi 74, 20157 Milan, Italy; 6 Department of Research, Advanced Diagnostics, and Technological Innovation, Translational Research Area, Regina Elena National Cancer Institute IRCCS, Istituti Fisioterapici Ospitalieri (IFO), via Elio Chianesi, 53 00144 Rome, Italy; 7 Cutaneous Physiopathology Lab, San Gallicano Dermatologic Institute, IRCCS, Istituti Fisioterapici Ospitalieri (IFO), via Elio Chianesi, 53 00144 Rome, Italy

## Abstract

**Background:**

Infective endocarditis (IE) is associated with high rates of mortality. Prolonged treatments with high-dose intravenous antibiotics often fail to eradicate the infection, frequently leading to high-risk surgical intervention. By providing a mechanism of antibiotic tolerance, which escapes conventional antibiotic susceptibility profiling, microbial biofilm represents a key diagnostic and therapeutic challenge for clinicians. This study aims at assessing a rapid biofilm identification assay and a targeted antimicrobial susceptibility profile of biofilm-growing bacteria in patients with IE, which were unresponsive to antibiotic therapy.

**Results:**

*Staphylococcus aureus* was the most common isolate (50%), followed by *Enterococcus faecalis* (25%) and *Streptococcus gallolyticus* (25%). All microbial isolates were found to be capable of producing large, structured biofilms in vitro. As expected, antibiotic treatment either administered on the basis of antibiogram or chosen empirically among those considered first-line antibiotics for IE, including ceftriaxone, daptomycin, tigecycline and vancomycin, was not effective at eradicating biofilm-growing bacteria. Conversely, antimicrobial susceptibility profile of biofilm-growing bacteria indicated that teicoplanin, oxacillin and fusidic acid were most effective against *S. aureus* biofilm*,* while ampicillin was the most active against *S. gallolyticus* and *E. faecalis* biofilm, respectively.

**Conclusions:**

This study indicates that biofilm-producing bacteria, from surgically treated IE, display a high tolerance to antibiotics, which is undetected by conventional antibiograms. The rapid identification and antimicrobial tolerance profiling of biofilm-growing bacteria in IE can provide key information for both antimicrobial therapy and prevention strategies.

## Background

Infective endocarditis (IE) is associated with a poor prognosis and a reduced life expectancy [1–4]. The mortality rate for patients with IE is approximately 25%, with more than one-third of patients dying within a year [5–8]. The rapid identification of the specific microbial etiology and targeted antimicrobial therapy are fundamental for optimal patient treatment [9, 10]. Blood culture represents the standard test to determine the microbial etiology of IE, providing identification for almost all cultivable species responsible for endocarditis, including *Candida* species [11]. However, negative blood cultures are frequent, thus contributing to diagnostic uncertainty [12–15]. In suspected cases, skin examination may provide important indicators to support a diagnostic suspect of IE [16–18]. Staphylococci, streptococci and enterococci are leading causes of IE, accounting for more than 70% of cases [1, 10]. In particular, *Staphylococcus aureus* is the most prevalent microbial isolate, associated with approximately 30% of cases of IE, while coagulase-negative staphylococci account for more than 10% of cases [5, 11, 19]. *Streptococci* account for 30% of IE. Among them, *Streptococcus gallolyticus* appears as the major causative agent, accounting for 20–50% of streptococcal-related IE [5, 11, 19]. *Enterococcus* is the third major cause of both native and prosthetic valve IE, with *Enterococcus faecalis* emerging as the most frequent species (10% of IE) [5, 11, 19, 20]. Conversely, gram-negative bacilli are rare, accounting for 5% of cases of IE, while Fungi have been only seldom described [11].

The marked IE pathogenicity relies on the ability of microorganisms to adhere, colonize and chronically persist on native or prosthetic valves, forming septic vegetations which consist of fibrin, platelets, inflammatory cells, and bacteria embedded within a structured biofilm matrix [15, 21, 22]. The mechanisms adopted by bacteria to form biofilms greatly differ among species and environmental conditions. Although the composition and function of the biofilm matrix can vary, some elements remain conserved. For instance, all biofilms contain extracellular polymeric substances (EPS) which mainly consists of polysaccharides, extracellular DNA, proteins and lipids, that holds bacterial cells together [22]. Compelling data from clinical and in vitro studies indicate that, by supporting adherence and colonization as well as increased tolerance to antibiotics and host immune defenses, microbial biofilms represent a key element in the pathogenesis of endocarditis, being associated with poor treatment outcomes and the frequent need of surgical intervention [8, 23–25].

This study was aimed at measuring biofilm production and antimicrobial susceptibility profile of biofilm-growing bacteria from patients with surgically-treated IE, to support clinical decision-making and more effective therapeutic and preventative strategies.

## Results

The study included eight patients who underwent cardiac surgery for IE caused by Gram-positive bacteria, with a vegetation size on echocardiography ≥1.2 cm. The diagnosis of IE was confirmed by transesophageal echocardiogram (TEE) echocardiography following the modified Duke Criteria [26]. The IE was localized on the aortic valve in 50.0% (4/8) of the patients, on the mitral valve in 37.5% (3/8), and on the mitro-aortic valves in 12.5% (1/8). The major predisposing factors were identified in previous surgical interventions (62.5%), followed by cardiac disease (37.5%), pulmonary disease (25.0%), HCV infection (25%) and hypertension (25%). Additional data on the underlying conditions administered treatment and clinical outcome are summarized in Table 1.

**Table 1:** Demographic and clinical data of the 8 patients with IE

Patients with endocarditis (*n* = 8)	
Male/Female	5/3
Age, years [median (range)]	57 (37–81)
Death due to IE	0
Vegetation cm [median (range)]	1.55 (1.2–2.2)
Aortic valve	4/8
Mitral valve	3/8
Mitro-aortic valve	1/8
Previous surgical interventions	5
Cardiac disease	3
Pulmonary disease	2
HCV	2
Hypertension	2
Esophagitis	1
Rheumatic polymyalgia	1
Rheumatoid arthritis	1
Acute kidney failure	1
Type 2 diabetes mellitus	1
Brain hemorrhage	1
Raynaud syndrome	1

Blood cultures gave a positive microbial identification in six cases, while in the remaining two cases, they were repeatedly negative (Table 2). Based on microbial identification and antimicrobial resistance profiles, patients received targeted antibiotic therapy (Table 2). Blood culture-negative patients, but with clinical evidence of IE (N1 and N7), were empirically treated as detailed in Table 2. In particular, antimicrobial regimens containing β-lactams were administered in all patients, Vancomycin was administered in 4 patients, gentamicin administered in 2 patients and linezolid in 1 case. In 89.9% (7/8) of cases, a combination regimen of 2 or more antibiotics was used. The antibiotic therapy was administered for a period of 2 to 6 weeks. Despite antimicrobial therapy, all patients underwent cardiac surgery. Following surgery and the removal of the native valves and the vegetations, samples were sent to the microbiological laboratory for further assessment. The results indicated that the direct culture method, without sonication, allowed microbial identification in 1/8 (12.5%) of samples. Conversely, specimen sonication allowed microbial isolation and identification in all samples, with significant improvement (*P* = 0.004) as compared to conventional methods. Notably, sonication allowed an etiological diagnosis also in the two patients with repeatedly negative blood cultures (Table 2). *S. aureus* was the most frequently isolated pathogen, present in 4 cases, one of which was identified as methicillin-resistant *S. aureus* (MRSA). *E. faecalis* and *S. gallolyticus* were both isolated in 2 cases (Table 2).

**Table 2:** Microbiologic Tests for bacterial isolation, antibiotic therapy and biofilm production. The level of biofilm production was measured by the cBRT

Patients	Blood culture before surgery	Hearth valve culture	Hearth valve sonication	Antibiotic therapy	Biofilm production	Vegetation (Cm)
N1	Negative	Negative	*S. aureus*	Ceftriaxone; Piperacillin/Tazobactam	High	1.4
N2	MRSA	MRSA	MRSA	Linezolid; Piperacillin/Tazobactam	Moderate	1.2
N3	*S. gallolyticus*	Negative	*S. gallolyticus*	Ceftriaxone; Gentamicin	Moderate	1.6
N4	*S. aureus*	Negative	*S. aureus*	Vancomycin; Meropenem	High	1.5
N5	*E. faecalis*	Negative	*E. faecalis*	Piperacillin-Tazobactam; Ampicillin; Meropenem	High	2.1
N6	*S. gallolyticus*	Negative	*S. gallolyticus*	Piperacillin-Tazobactam; Vancomycin	High	2.2
N7	Negative	Negative	*S. aureus*	Ceftriaxone; Vancomycin; Piperacillin/Tazobactam	Moderate	1.3
N8	*E. faecalis*	Negative	*E. faecalis*	Vancomycin	High	1.8

### Assessment of the antimicrobial resistance profiles

The antibiotic susceptibility profiles were determined according to EUCAST clinical breakpoint tables v 9.0. The results show that all the strains appeared susceptible to vancomycin (Table 3). *S. aureus* was highly susceptible to almost all tested antibiotics, but 100% resistant to benzylpenicillin. *E. faecalis* were susceptible to ampicillin, ampicillin/sulbactam, linezolid, teicoplanin, and tigecycline but resistant to clindamycin, erythromycin, and trimethoprim/sulfamethoxazole. Finally, *S. gallolyticus* isolates were susceptible to all antibiotics tested (Table 3).

**Table 3:** Antibiotic resistance profile (% of resistance) of *S. aureus, E. faecalis* and *S. gallolyticus* (% of resistance) strains, obtained by the Antimicrobial Susceptibility Testing (AST) and the anti-biofilm test (ABT). N represents the number of samples. Ampicillin/Sulbactam (AMP/SUL), High-level gentamicin (HLG), High-level streptomycin (HLS), Trimethoprim/Sulfamethoxazole (TMP/SMX)

Drug	AST			ABT		
	*S. aureus* (*N* = 4)	*E. faecalis* (*N* = 2)	*S. gallolyticus* (N = 2)	*S. aureus* (N = 4)	*E. faecalis* (N = 2)	*S. gallolyticus* (N = 2)
Ampicillin	–	0	0	–	0	0
AMP/SUL	–	0	–	–	0	–
Benzilpenicillin	100	–	0	100	–	50
Cefotaxime	–	–	0	–	–	50
Ceftriaxone	–	–	0	–	–	100
Clindamycin	25	100	0	75	100	100
Daptomycin	0	–	–	100	–	–
Erythromycin	25	100	–	100	100	–
Fusidic Acid	0	–	–	25	–	–
Gentamicin	0	–	–	50	–	–
HLG	–	50	–	–	100	–
Levofloxacin	25	–	–	100	–	–
Linezolid	0	0	–	100	50	–
Oxacillin	25	–	–	25	–	–
Rifampicin	0	–	–	50	–	–
HLS	–	50	–	–	100	–
Teicoplanin	0	0	–	0	50	–
Tigecyclin	0	0	–	100	100	–
TMP/SMX	0	100	–	75	100	–
Vancomycin	0	0	0	100	100	50

### Quantification of biofilm production

Biofilm production for each bacterial isolate was assessed by the clinical Biofilm Ring Test (cBRT) [27]. The results showed that *S. aureus* isolates were moderate (2/4 cases, including the MRSA) or high (2/4 cases) biofilm-producers. *E. faecalis* strains were both high biofilm producers as well as the *S. gallolyticus* strains, which were found to be moderate/high biofilm producers (Table 2). Thus, all the bacterial strains were classified in the range of moderate/high biofilm-producers.

**Fig. 1:**
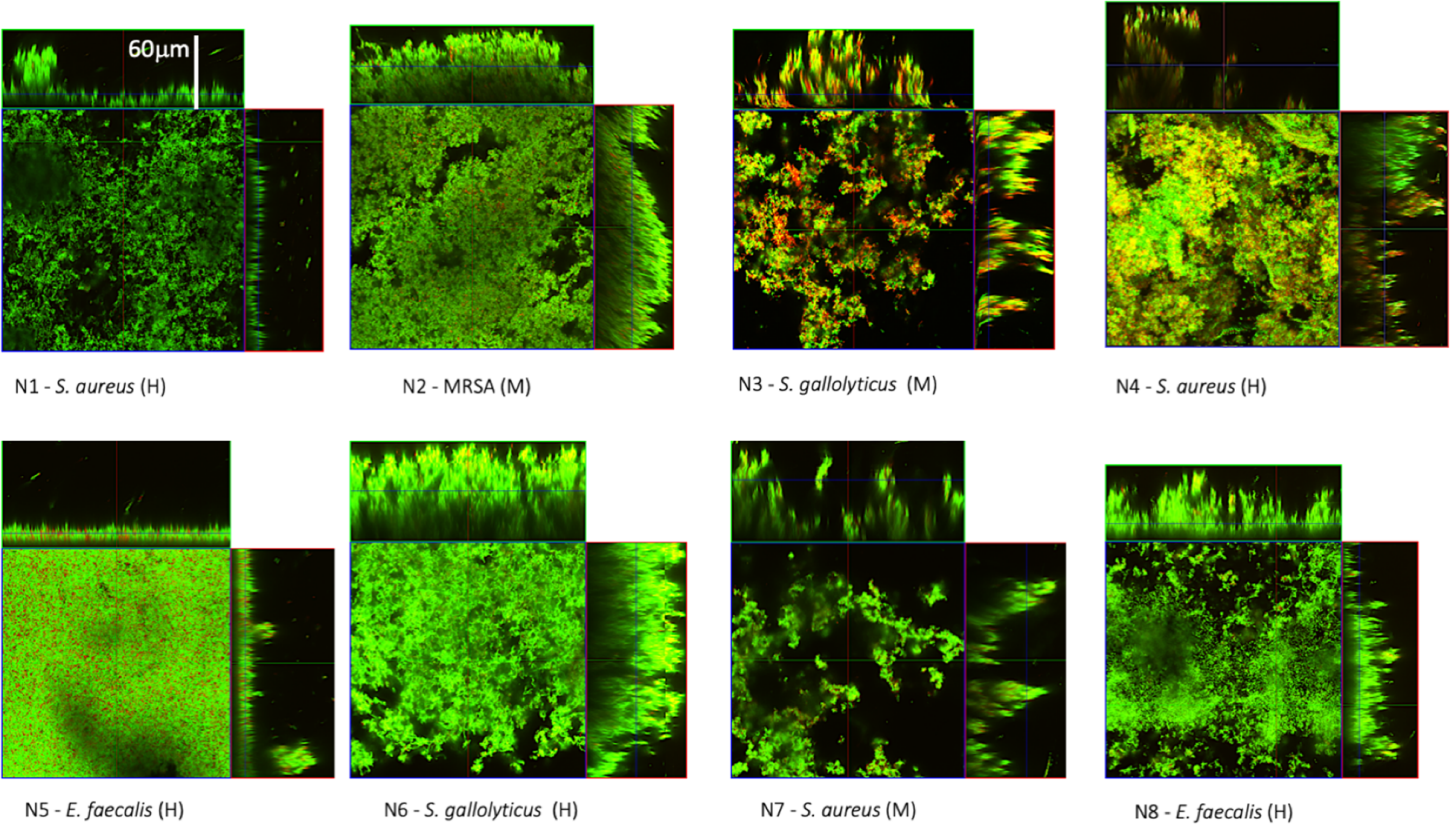
Confocal microscopy analysis of biofilms. Representative images of biofilms developed on polystyrene pegs following 24 h incubation at 37 °C. Green indicates viable bacteria, and red dead bacteria. Orthogonal sections displaying horizontal (*z*) and side views (*x* and *y*) of reconstructed 3D biofilm images are shown. The eight bacterial isolates (N1–8) are classified into Moderate (M) or High (H) biofilm-producers according to cBRT

Confocal microscopy analysis of the biofilms (Fig. 1), examined after 24 h of incubation, was highly consistent with cBRT biofilm assessment. All the strains gave a full coverage throughout the entire extension of the substrate with the development of a thick and structurally complex biofilm of approximately 60 μm in height, with a marked predominance of live (green) cells.

### Antibiotic susceptibility of biofilm-producing isolates

The susceptibility profiles gathered by conventional antimicrobial susceptibility testing were apparently in contrast to the lack of response to the antimicrobial therapy in vivo. Since all of the strains were substantial biofilm producers, we next evaluated whether the ability to produce biofilm might correlate with the increased resistance to antibiotics. To this end, the antimicrobial susceptibility profiles were assessed in microbial isolates growing in a biofilm. As for the AST, the ABT was performed by the broth dilution-based procedure, to ensure the comparability of the data. The results are summarized in Table 3. The antibiotic susceptibility of the different strains growing in biofilm gave resistance profiles which greatly differed from those gathered by AST. In particular, only 30.0% of the antibiotics tested were found to be in the range of the susceptibility breakpoint. Statistical analyses confirmed a significant difference of the overall antimicrobial susceptibility profiles by comparing the data gathered by conventional susceptibility testing to those obtained in biofilm-growing bacteria (*P* < 0.001).

All biofilm-growing *E. faecalis* and *S. gallolyticus* were susceptible to ampicillin, while the 3 methicillin-sensitive *S. aureus,* confirmed their susceptibility to oxacillin also when tested by ABT. Teicoplanin was effective against *E. faecalis* and *S. aureus* biofilms in 5 isolates out of 6 (83.3%), while 3 out of 4 isolates of *S. aureus* were susceptible to fusidic acid. Conversely, almost all the isolates were resistant to ceftriaxone, daptomycin, erythromycin, high level gentamicin, levofloxacin, linezolid, tigecycline trimethoprim/sulfamethoxazole and vancomycin. The spectrum of antimicrobial efficacy on different biofilm-growing bacterial isolates revealed that *S. aureus* was susceptible to 23.1–50.0% of the tested antibiotics, while *E. faecalis* and *S. gallolyticus* gave results below the breakpoints in only 33.3–50.0% of the antibiotics (Additional file 2). Notably, *S. aureus* N4, *S. gallolyticus* N6, *S. aureus* N7 and *E. faecalis* N8, which were unsuccessfully cleared by treatment with vancomycin in vivo*,* were resistant to this antibiotic when grown in biofilm. Likewise, the MRSA N2 and *S. gallolyticus* N3 strains, treated with linezolid and ceftriaxone, respectively, in vivo, were found to be tolerant to these antibiotics when tested in a biofilm. In the absence of positive blood culture, patient N1 was empirically treated with ceftriaxone and piperacillin/tazobactam. Therefore, for the *S. aureus* strain, subsequently isolated by sonication, comparative analysis between AST and ABT was not possible. *E. faecalis* N5, which was treated by ampicillin in vivo, was the only bacterial isolate found susceptible by both AST and ABT.

## Discussion

IE is a severe multisystem disease with mortality rates of approximately 25% and a generally poor response to antimicrobial therapy [8]. Successful eradication is difficult to achieve and relapse is common even after a prolonged antimicrobial therapy [8]. Possible causes include a limited or compromised host response against biofilms, as well as the relatively high bacterial density within vegetations [8, 23–25]. In the present study, the echocardiographic findings revealed the presence of vegetations (≥ 1.2 cm) in all patients located on the aortic valve (4 patients, 50%), mitral valve (3 patients, 37.5%) and mitro/aortic valve (1 patient, 12.5%).

The most frequent bacterial etiology was represented by *S. aureus*, which was isolated from 50% of the samples, followed by *E. faecalis* and *S. gallolyticus,* both isolated in 2 cases. This finding is consistent with previous studies showing that staphylococci, streptococci, and enterococci, are responsible for approximately 80% of IE cases [28–35]. Conventional antimicrobial susceptibility assessment showed that all the bacterial isolates were highly susceptible in vitro to most antibiotics, including those considered of choice for the treatment of IE. In particular, all of the clinical isolates appeared susceptible to vancomycin, which is the antibiotic of choice in the treatment of MRSA and a first-line therapy against enterococci and streptococci [8, 10]. *S. aureus* isolates were all susceptible to daptomycin, fusidic acid, gentamicin, linezolid, rifampicin, teicoplanin, tigecycline and trimethoprim/sulfamethoxazole but were found to be 100% resistant to benzylpenicillin. MRSA was the causative agent of one case of IE (12.5%), and it was found to be resistant to benzylpenicillin, oxacillin and levofloxacin. *E. faecalis* isolates were susceptible to ampicillin, ampicillin/sulbactam, linezolid, teicoplanin, tigecycline, while *S. gallolyticus* isolates were found to be susceptible to all the antibiotics tested (Table 3). In all cases, the administered antibiotic therapy failed to achieve complete eradication of bacteria, leading to a clinical relapse and the need for surgical treatment. The high bacterial density in a structured biofilm matrix within the vegetation can pose a physical and metabolic barrier which might, at least in part, explain this failure. The assessment of biofilm production by the cBRT and confocal microscopy analysis showed that all the clinical isolates were able to readily develop a highly adhesive and structurally complex biofilm matrix, being therefore classified as moderate/high biofilm producers. Notably, none of the bacterial isolates was found to be weak biofilm producer. The images collected by confocal microscopy were highly consistent with cBRT measurements, revealing that the moderate/high biofilm production as measured by cBRT gave rise to a compact biofilm matrix of approximately 60 μm in height with all microbial isolates (Fig. 1).

It has been suggested that the antibiotic concentration required to eradicate biofilm-related infections can be a hundred times higher than the MIC for the same microorganism as assessed in planktonic culture by conventional antibiograms [36–38]. In this study, biofilm-growing microbial cultures were found to be highly tolerant to antibiotics as compared to their planktonic counterparts, which is usually explored by the conventional antimicrobial profiling (*P* < 0.001). *S. aureus* N4, *S. gallolyticus* N6, *S. aureus* N7 and *E. faecalis* N8, which were challenged with vancomycin in vivo, showed a poor susceptibility to this antibiotic when assessed in a mature biofilm. Likewise, the MRSA N2 and *S. gallolyticus* N3 strains, unsuccessfully challenged in vivo with linezolid and ceftriaxone, respectively, on the basis of the antibiogram, were found to be tolerant to these antibiotics when tested in biofilm. The *S. aureus* N1 was empirically, and unsuccessfully treated in vivo with ceftriaxone and piperacillin/tazobactam and only after surgery was subjected to a 4 weeks regimen of gentamicin, to which it was found susceptible by both AST and ABT. *E. faecalis* N5, which was challenged in vivo with ampicillin, was the only bacterial isolate which was found susceptible to the administered antibiotic by both AST and ABT. Failure of this antibiotic at eradicating *E. faecalis* N5 can be reasonably attributable to the very short duration of the antibiotic therapy (6 days).

Overall, the most effective antibiotics against biofilm-growing *S. aureus* were fusidic acid, oxacillin and Teicoplanin, with MBEC values below breakpoints in almost all isolates, while ampicillin and ampicillin/sulbactam were the most active drugs in IE caused by both *E. faecalis* and *S. gallolyticus,* respectively. This latter data is in agreement with previous observations, confirming the effectiveness of ampicillin against ampicillin-susceptible *E. faecalis*, particularly in those patients with aminoglycoside resistance, or at risk of aminoglycoside-nephrotoxicity [7, 8, 39]. Indeed, aminoglycosides are no longer recommended in the treatment of staphylococcal and enterococcal native valve endocarditis due to their limited clinical benefits, the increasing frequency of microbial resistance and renal toxicity [34, 40]. Gentamicin, which was found to be effective against planktonic-growing *S. aureus,* according to AST, gave minimal biofilm-eradication concentration (MBEC) values below breakpoints only in 50% of isolates. Besides, gentamicin and streptomycin at high dosage showed a poor efficacy at eradicating biofilm-growing *E. faecalis*, which appeared to be susceptible to both drugs by conventional AST. Short term therapy (2 weeks) with gentamicin in combination with ceftriaxone has been previously suggested for the treatment of uncomplicated *S. gallolyticus* endocarditis [41–43]. Indeed, *S. gallolyticus* strains appeared susceptible to ceftriaxone by AST, but they were found to be resistant when assessed in the biofilm matrix.

Rifampicin is recommended as an adjunctive treatment in prosthetic valve endocarditis caused by staphylococci. However, additional clinical evidence is necessary to support the addition of rifampicin in the treatment of staphylococcal native valve endocarditis and in surgically treated patients [10, 44]. In the present study, rifampicin was found to be effective against 50% of *S. aureus* strains grown in biofilm. Indeed, rifampicin was active only against moderate biofilm-producers but failed against high biofilm-producing *S. aureus*. The poor efficacy of rifampicin against a structured microbial biofilm was also observed in previous studies reporting only a limited bacterial killing against high biofilm-producing *S. aureus* [36–45].

High-dose daptomycin (10 mg/kg/day) alone or in combination with a second antibiotic, is considered a viable alternative to vancomycin for the treatment of staphylococcal endocarditis [10, 46–48]. However, the failure of daptomycin in patients with bacteremia and endocarditis caused by *S. aureus* leading to persistent or relapsing infection was previously reported [49]. In this study, daptomycin had a poor efficacy against biofilm-producing *S. aureus* strains (Table 3). These results are in agreement with previous studies reporting a limited daptomycin efficacy at eradicating biofilm-related infections in vitro and animal models [24, 50–58]. Daptomycin induces a dual action on both the cell membrane and cell wall, creating ion channels that lead to bacterial death [59, 60]. Thus, direct access to the bacterial cell wall is a necessary prerequisite for daptomycin anti-microbial activity [61, 62]. The large, highly structured biomass produced by IE microbial isolates may present a physical barrier to the free drug diffusion, thus limiting daptomycin activity.

Vancomycin is considered a standard treatment for in IE sustained by MRSA, and, more generally, an appropriate empiric choice against Gram-positive bacteria [8, 63], although clinical failures and poor penetration into vegetations have been frequently reported [10, 64]. Indeed, all the strains appeared susceptible to vancomycin by conventional profiling of planktonic growing bacteria. However, drug susceptibility assessment of all the 8 clinical isolates in mature biofilms, gave resistance profiles strikingly different from those gathered in planktonic cultures, showing MBEC values below breakpoints only with the *S. gallolyticus* strain, which was classified as a moderate biofilm producer. The large size and complex structure of the vancomycin molecule might again play a part in the reduced accessibility of bacterial cells within the biofilm both in vivo and in vitro [62, 64]. The result of this study reinforces previous evidence of a poor vancomycin efficacy against biofilm growing Gram-positive bacteria in IE [62, 64]. In contrast, teicoplanin, which is another member of the glycopeptide family of antibiotics, ranked as the most effective drug against biofilm-growing *S. aureus*, showing MBEC values below breakpoints in all isolates (Table 3 and Additional file 2). This is in line with previous studies suggesting teicoplanin as a possible alternative therapeutic option to vancomycin in IE and for patients harboring MRSA and presenting a limited renal function [65, 66].

Early identification of the causative microbial agent and its drug susceptibility profile are crucial in IE, since a delayed antimicrobial treatment negatively affects the clinical outcome [6, 67]. However, microbial isolation and identification remain a challenge in IE, as blood cultures may yield false-negative results in 2.5 to 70% of all cases of endocarditis, particularly among those patients with prior antimicrobial therapy [10, 11, 34, 68, 69]. In this study, blood cultures allowed bacterial isolation in 6 (75%) cases, while in the other two patients (25%), it gave negative results. To improve the chance of a positive bacterial isolation, a sonication procedure was applied to the surgically removed heart valve tissue samples before culture plating. This procedure allowed isolation of pathogenic microorganisms in all samples, thus significantly improving (*P* = 0.004) the assay sensitivity, which by conventional methods allowed microbiological diagnosis in only one case (12.5%). Although gathered in a relatively small group of patients, these results show that by dispersing microbial cells from the tissue/biofilm matrix, sonication can increase significantly the probability to isolate microbial pathogens even in blood culture-negative endocarditis [18, 70, 71]. Even considering that all patients received antibiotic therapy before surgery and tissue collection, the very low sensitivity of the direct tissue culture as compared to the blood culture is intriguing as well as the significant difference observed between the direct tissue culture and sonication. Indeed, the increased sensitivity allowed by the sonication-based procedure may further represent indirect evidence of the presence of biofilm-growing or tightly adherent pathogens, in the valve tissue vegetation [10, 22, 34, 72].

## Conclusions

IE therapy requires a prolonged administration of antibiotics [8]. The positive therapeutic outcome depends on multiple factors, including the location and size of the vegetation, patient comorbidities and surgical intervention [49, 63]. The specific cause of most failures of antibiotic therapy is still unclear. Growing evidence suggests that the ability to produce biofilms may play a major pathogenetic role in supporting microbial adhesion and persistence while protecting from antimicrobial drugs [22, 73, 74]. Our results support the notion that IE represents an example of a biofilm-related infection, which, in turn, is associated with an increased antibiotic tolerance. Surgical disruption and removal of microbial vegetation can largely remove the biofilm structure, thus improving patients’ healing. Although this study presents some limitations, due to the small group of highly selected patients and the relatively short period of follow-up, the results strongly point to the need of pursuing novel strategies to allow for early microbiological diagnosis and effective antibiotic therapy against endocarditis sustained by biofilm-growing bacteria. A sonication-based culture method appears as a necessary procedure to allow the detection of a higher proportion of latent microorganisms encased in the biofilm structure. Further, measurement of the biofilm production in blood-culture positive cases may offer a useful biomarker to predict the clinical and therapeutic outcomes. Indeed, the cBRT represents a suitable diagnostic system for most microbiology laboratories. Moreover, the antibiotic susceptibility profile of biofilm-growing bacteria should always be assessed, in addition to standard AST, since it may offer key susceptibility information, particularly in the case of invasive IE, where biofilm represents a recognized pathogenic element.

## Methods

### Patient’s recruitment and clinical investigation

Eight patients with IE, defined according to the modified Duke criteria [26], were recruited at the Hospital Cardiosurgery Unit during 2016. The epidemiological and clinical data, as well as the therapeutic interventions for each patient, are summarized in Table 1. The presence of IE was confirmed in all patients by transthoracic and transesophageal echocardiography. Blood cultures were performed before antibiotic therapy in all patients enrolled in the study.

### Microbiological diagnosis

Blood cultures from each patient were assessed in the Laboratory of Clinical Microbiology, Virology and Bioemergencies – ASST Fatebenefratelli-Sacco using the BacT ALERT 3D (Biomerieux, Marcy-l’Etoile, France) automatized blood culture system. Microbial identification was performed by matrix-assisted laser desorption/ionisation – time of flight mass spectrometry (MALDI-TOF MS system – Bruker Daltonics, Bremen, Germany) and by sequence analysis (ABI PRISM 3130xl Genetic Analyzer) of the 16S rRNA gene [75]. Antimicrobial susceptibility testing (AST) was performed by the Vitek 2.0 system (Biomerieux, Marcy-l’Etoile, France) and by the broth microdilution test (Thermo Scientific, Massachusetts, USA) for the definition of the Minimum Inhibitory Concentration (MIC) criteria, according to the European Committee on Antimicrobial Susceptibility Testing (EUCAST clinical breakpoint table v 9.0). The range of antibiotics tested is listed in the additional files section (Additional file 1). Strains were classified as MRSA when presenting both oxacillin resistance (MIC ≥4 mg/ml) and positive agglutination test for Penicillin-Binding Protein (PBP2, Oxoid, Basingstoke, UK) [76].

### Specimen collection

In the operating room, the explanted heart valves were removed aseptically placed in sterile tubes and transported to the microbiology laboratory within 1 h. The specimen was then portioned in two parts, one was readily cultured without sonication, whereas the other portion was cultured after sonication as described below. Direct culture: Specimen was placed in Castaneda flasks (DID, Italy), incubated at 37 °C, and subcultured daily in suitable growth medium (Columbia agar + 5% sheep blood, Mac Conkey agar, Sabouraud agar, Mannitol salt agar, and Schaedler agar + 5% sheep blood; (Biomerieux, Marcy-l’Etoile, France) [77]. Aerobic and anaerobic agar plates were incubated at 37 °C for 5 days, and the microorganisms were identified using conventional methods.

### Sonication

Specimens were placed in separate sterile containers and sterile normal saline was added to cover the sample completely. After vortexing for 30s, the sample was sonicated by use of an ultrasound bath (VWR, Milan, Italy) for 5 min at a frequency of 30 kHz and vortexed for another 30s [78]. The outside of the container was treated with 70% ethanol. The resulting sonication fluid was centrifuged at 4000 rpm for 15 min, and the sediment was used for subsequent microbiological cultures onto chocolate agar, Columbia blood agar, Mannitol salt agar, MacConkey agar, Sabouraud agar and Schaedler agar plates (Biomerieux, Marcy-l’Etoile, France) and inoculated into Brain Heart Infusion and Thyoglycollate broths (TermoFhisher, Cornaredo, Italy). Anaerobic and aerobic agar plates were incubated at 37 °C for 5 days while broths were incubated for 15 days in the same conditions. Microorganisms were identified using conventional methods.

### Biofilm production

Biofilm production was analyzed by the clinical BioFilm Ring Test (cBRT) (Biofilm Control, Saint Beauzire, France) as previously described [27]. *S. aureus* strains ATCC 25923 and *Staphylococcus epidermidis* ATCC 12228 were included in each plate as standard reference and internal control. Each strain was analyzed in duplicate and experiments were repeated 3 times.

### Biofilm imaging

Bacterial colonies, grown overnight on blood agar plates, were inoculated into 3 ml of 0.45% saline solution (Air Life, Carefusion, CA, USA) to obtain a turbidity of 0.5 ± 0.1 McFarland (McF) corresponding approximately to 1 × 10^8^ colony-forming units (CFU)/ml. Samples were diluted 1:1000 and resuspended in 1 ml of brain heart infusion broth (BHI) in a μ-Slide, 8 well glass bottom chamber slides (Ibidi, Germany). The bacterial suspension was incubated at 37 °C for 24 h to allow biofilm formation. Afterwards, the medium was removed and biofilms were washed with 0.45% saline solution. The samples were stained using the LIVE/DEAD BacLight kit (Life Technologies, New York, NY, USA), according to supplier specifications. Biofilm samples were analyzed using a Zeiss LSM5 Pascal Laser Scan Microscope (Zeiss, Oberkochen, Germany) as described previously [36].

### Antimicrobial susceptibility of bacterial isolates in biofilm

The anti-biofilm test (ABT) was performed by the protocol previously described in Di Domenico et al. [36].

### Statistics

Statistical analysis was performed using the chi-square test when appropriate. Observed differences were considered statistically significant, with *p*-values of 0.05 or less [36].

## Supplementary information

**Additional file 1:.** List and concentration range (μg/ml) of the antibiotics tested. (PPTX 43 kb)

**Additional file 2: **Comparison between the Antimicrobial susceptibility test (AST) and the Anti-Biofilm Test (ABT). Susceptibility (S) and Resistance profiles of *S. aureus* (N1, N2, N4 and N7), *E. faecalis* (N5 and N8) and *S. gallolyticus* (N3 and N6) clinical isolates to different antimicrobials. Classification was performed according to the European Committee on Antimicrobial Susceptibility Testing clinical breakpoint tables (EUCAST clinical breakpoint table v 9.0). Ampicillin/Sulbactam (AMP/SUL), HLG - High level gentamicin, HLS - High level streptomycin, TXP/SMX - Trimethoprim/Sulfamethoxazole. (TIFF 5897 kb)

## Data Availability

The datasets used and/or analyzed during the current study are available from the corresponding author on reasonable request.

## Abbreviations

ABT: Anti-biofilm test
AMP/SUL: Ampicillin/sulbactam
AST: Antimicrobial susceptibility testing
cBRT: Clinical Biofilm Ring Test®
CFU: Colony-forming unit
HLG: High-level gentamicin, HLS: high-level streptomycin
IE: Infective endocarditis
MBEC: Minimal biofilm-eradication concentration
MIC: Minimum inhibitory concentration
MRSA: Methicillin-resistant *Staphylococcus aureus*
TMP/SMX: Trimethoprim/sulfamethoxazole

## References

[1] Pant S, Patel NJ, Deshmukh A, Golwala H, Patel N, Badheka A, Hirsch GA, Mehta JL (2015). Trends in infective endocarditis incidence, microbiology, and valve replacement in the United States from 2000 to 2011. J Am Coll Cardiol.

[2] Baddour LM, Wilson WR, Bayer AS, Fowler VG, Tleyjeh IM, Rybak MJ (2015). Infective endocarditis in adults: diagnosis, antimicrobial therapy, and management of complications: a scientific statement for healthcare professionals from the American Heart Association. Circulation..

[3] Gatti G, Benussi B, Gripshi F, Della Mattia A, Proclemer A, Cannatà A (2017). A risk factor analysis for in-hospital mortality after surgery for infective endocarditis and a proposal of a new predictive scoring system. Infection.

[4] Østergaard L, Valeur N, Wang A, Bundgaard H, Aslam M, Gislason G, et al. Incidence of infective endocarditis in patients considered at moderate risk. Eur Heart J. 2018. 10.1093/eurheartj/ehy629. 10.1093/eurheartj/ehy629 30346503

[5] Murdoch DR, Corey GR, Hoen B, Miró JM, Fowler VG, Bayer AS (2009). International collaboration on endocarditis-prospective cohort study (ICE-PCS) investigators. 2009. Clinical presentation, etiology, and outcome of infective endocarditis in the 21st century: the international collaboration on endocarditis-prospective cohort study. Arch Intern Med.

[6] Thuny F, Grisoli D, Collart F, Habib G, Raoult D (2012). Management of infective endocarditis: challenges and perspectives. Lancet..

[7] Fernández-Hidalgo N, Tornos Mas P (2013). Epidemiology of infective endocarditis in Spain in the last 20 years. Rev Esp Cardiol.

[8] Holland TL, Baddour LM, Bayer AS, Hoen B, Miro JM, Fowler VG (2016). Infective endocarditis. Nat Rev Dis Primers.

[9] Fukuchi T, Iwata K, Ohji G (2014). Failure of early diagnosis of infective endocarditis in Japan--a retrospective descriptive analysis. Medicine (Baltimore).

[10] Habib G, Lancellotti P, Antunes MJ, Bongiorni MG, Casalta JP, Del Zotti F, et al. 2015 ESC Guidelines for the management of infective endocarditis: The Task Force for the Management of Infective Endocarditis of the European Society of Cardiology (ESC). Endorsed by: European Association for Cardio-Thoracic Surgery (EACTS), the European Association of Nuclear Medicine (EANM). Eur Heart J. 2015;36:3075–3128. 10.1093/eurheartj/ehv319 26320109

[11] Liesman RM, Pritt BS (2015). Maleszewski JJ, Patel R. 2017. Laboratory diagnosis of infective endocarditis. J Clin Microbiol.

[12] Nihoyannopoulos P, Oakley CM, Exadactylos N, Ribeiro P, Westaby S, Foale RA (1985). Duration of symptoms and the effects of a more aggressive surgical policy: two factors affecting prognosis of infective endocarditis. Eur Heart J.

[13] Lodise TP, McKinnon PS, Swiderski L, Rybak MJ (2003). Outcomes analysis of delayed antibiotic treatment for hospital-acquired Staphylococcus aureus bacteremia. Clin Infect Dis.

[14] Dickerman SA, Abrutyn E, Barsic B, Bouza E, Cecchi E, Moreno A (2003). The relationship between the initiation of antimicrobial therapy and the incidence of stroke in infective endocarditis: an analysis from the ICE prospective cohort study (ICE-PCS). Am Heart J.

[15] Long B, Koyfman A (2018). Infectious endocarditis: an update for emergency clinicians. Am J Emerg Med.

[16] Servy A, Valeyrie-Allanore L, Alla F, Lechiche C, Nazeyrollas P, Chidiac C (2014). Prognostic value of skin manifestations of infective endocarditis. JAMA Dermatol.

[17] Tikoo M, Bardia A, Gupta A, Pandey A (2015). Dermatological manifestations of infective endocarditis. J Gen Intern Med.

[18] Gomes RT, Tiberto LR, Bello VN, Lima MA, Nai GA, Abreu MA (2016). Dermatologic manifestations of infective endocarditis. An Bras Dermatol.

[19] Raoult D, Casalta JP, Richet H, Khan M, Bernit E, Rovery C, Branger S, Gouriet F, Imbert G, Bothello E, Collart F, Habib G (2005). Contribution of systematic serological testing in diagnosis of infective endocarditis. J Clin Microbiol.

[20] Pericàs JM, Cervera C, Moreno A, Garcia-de-la-Mària C, Almela M, Falces C (2018). Outcome of Enterococcus faecalis infective endocarditis according to the length of antibiotic therapy: preliminary data from a cohort of 78 patients. PLoS One.

[21] Jung CJ, Yeh CY, Shun CT, Hsu RB, Cheng HW, Lin CS (2012). Platelets enhance biofilm formation and resistance of endocarditis-inducing streptococci on the injured heart valve. J Infect Dis.

[22] Elgharably H, Hussain ST, Shrestha NK, Blackstone EH, Pettersson GB (2016). Current hypotheses in cardiac surgery: biofilm in infective endocarditis. Semin Thorac Cardiovasc Surg.

[23] Eng RH, Padberg FT, Smith SM, Tan EN, Cherubin CE (1991). Bactericidal effects of antibiotics on slowly growing and nongrowing bacteria. Antimicrob Agents Chemother.

[24] LaPlante KL, Rybak MJ (2004). Impact of high-inoculum Staphylococcus aureus on the activities of nafcillin, vancomycin, linezolid, and daptomycin, alone and in combination with gentamicin, in an in vitro pharmacodynamic model. Antimicrob Agents Chemother.

[25] Presterl E, Grisold AJ, Reichmann S, Hirschl AM, Georgopoulos A, Graninger W (2005). Viridans streptococci in endocarditis and neutropenic sepsis: biofilm formation and effects of antibiotics. J Antimicrob Chemother.

[26] Li JS, Sexton DJ, Mick N, Nettles R, Fowler VG, Ryan T (2000). Proposed modifications to the duke criteria for the diagnosis of infective endocarditis. Clin Infect Dis.

[27] Di Domenico EG, Toma L, Provot C, Ascenzioni F, Sperduti I, Prignano G, et al. Development of an in vitro assay, based on the BioFilm Ring Test®, for rapid profiling of biofilm-growing bacteria. Front Microbiol. 2016:7:1429. eCollection 2016. 10.3389/fmicb.2016.01429 PMC5030256 27708625

[28] Puls M, Eiffert H, Hünlich M, Schöndube F, Hasenfuß G, Seipelt R (2013). Prosthetic valve endocarditis after transcatheter aortic valve implantation: the incidence in a single center cohort and reflections on clinical, echocardiographic and prognostic features. Euro-Intervention..

[29] Latib A, Naim C, De Bonis M, Sinning JM, Maisano F, Barbanti M (2014). TAVR-associated prosthetic valve infective endocarditis: results of a large, multicenter registry. J Am Coll Cardiol.

[30] Olsen NT, De Backer O, Thyregod HG, Vejlstrup N, Bundgaard H, Søndergaard L (2015). Prosthetic valve endocarditis after transcatheter aortic valve implantation. Circ Cardiovasc Interv.

[31] Amat-Santos IJ, Messika-Zeitoun D, Eltchaninoff H, Kapadia S, Lerakis S, Cheema AN (2015). Infective endocarditis after transcatheter aortic valve implantation: results from a large multicenter registry. Circulation..

[32] Regueiro A, Linke A, Latib A, Ihlemann N, Urena M, Walther T (2016). Association between transcatheter aortic valve replacement and subsequent infective endocarditis and in hospital death. JAMA..

[33] Mangner N, Woitek F, Haussig S, Schlotter F, Stachel G, Höllriegel R (2016). Incidence, predictors, and outcome of patients developing infective endocarditis following transfemoral transcatheter aortic valve replacement. J Am Coll Cardiol.

[34] Cahill TJ, Baddour LM, Habib G, Hoen B, Salaun E, Pettersson GB (2017). Challenges in infective endocarditis. J Am Coll Cardiol.

[35] Ferraris L, Milazzo L, Ricaboni D, Mazzali C, Orlando G, Rizzardini G (2013). Profile of infective endocarditis observed from 2003 - 2010 in a single center in Italy. BMC Infect Dis.

[36] Di Domenico EG, Cavallo I, Bordignon V, Prignano G, Sperduti I, Gurtner A (2018). Inflammatory cytokines and biofilm production sustain Staphylococcus aureus outgrowth and persistence: a pivotal interplay in the pathogenesis of atopic dermatitis. Sci Rep.

[37] Girard LP, Ceri H, Gibb AP, Olson M, Sepandj F (2010). MIC versus MBEC to determine the antibiotic sensitivity of Staphylococcus aureus in peritoneal dialysis peritonitis. Perit Dial Int.

[38] Castaneda P, McLaren A, Tavaziva G, Overstreet D (2016). Biofilm antimicrobial susceptibility increases with antimicrobial exposure time. Clin Orthop Relat Res.

[39] Pericas JM, Cervera C, del Rio A, Moreno A, Garcia de la Maria C, Almela M (2014). Changes in the treatment of Enterococcus faecalis infective endocarditis in Spain in the last 15 years: from ampicillin plus gentamicin to ampicillin plus ceftriaxone. Clin microb infect.

[40] Cosgrove SE, Vigliani GA, Fowler VG, Abrutyn E, Corey GR, Levine DP (2009). Initial low-dose gentamicin for Staphylococcus aureus bacteremia and endocarditis is nephrotoxic. Clin Infect Dis.

[41] Francioli P, Etienne J, Hoigne R, Thys JP, Gerber A (1992). Treatment of streptococcal endocarditis with a single daily dose of ceftriaxone sodium for 4 weeks. Efficacy and outpatient treatment feasibility. JAMA..

[42] Francioli P, Ruch W, Stamboulian D (1995). Treatment of streptococcal endocarditis with a single daily dose of ceftriaxone and netilmicin for 14 days: a prospective multicenter study. Clin Infect Dis.

[43] Sexton DJ, Tenenbaum MJ, Wilson WR, Steckelberg JM, Tice AD, Gilbert D, et al. Ceftriaxone once daily for four weeks compared with ceftriaxone plus gentamicin once daily for two weeks for treatment of endocarditis due to penicillin-susceptible streptococci. Endocarditis Treatment Consortium Group. Clin Infect Dis 1998;27:1470–1474. 10.1086/515038 9868662

[44] Forrest GN, Tamura K (2010). Rifampin combination therapy for non mycobacterial infections. Clin Microbiol Rev.

[45] Cerca N, Gomes F, Pereira S, Teixeira P, Oliveira R (2012). Confocal laser scanning microscopy analysis of S. epidermidis biofilms exposed to farnesol, vancomycin and rifampicin. BMC Res Notes.

[46] Dhand A, Bayer AS, Pogliano J, Yang SJ, Bolaris M, Nizet V (2011). Use of antistaphylococcal beta-lactams to increase daptomycin activity in eradicating persistent bacteremia due to methicillin-resistant Staphylococcus aureus: role of enhanced daptomycin binding. Clin Infect Dis.

[47] Miro JM, Entenza JM, Del Río A, Velasco M, Castañeda X, Garcia de la Mària C (2012). Hospital clinic experimental endocarditis study group. High-dose daptomycin plus fosfomycin is safe and effective in treating methicillin-susceptible and methicillin-resistant Staphylococcus aureus endocarditis. Antimicrob Agents Chemother.

[48] Kullar R, Casapao AM, Davis SL, Levine DP, Zhao JJ, Crank CW (2013). A multicentre evaluation of the effectiveness and safety of high-dose daptomycin for the treatment of infective endocarditis. J Antimicrob Chemother.

[49] Fowler VG, Boucher HW, Corey GR, Abrutyn E, Karchmer AW, Rupp ME (2006). Daptomycin versus standard therapy for bacteremia and endocarditis caused by Staphylococcus aureus. N Engl J Med.

[50] Cotroneo N, Harris R, Perlmutter N, Beveridge T, Silverman JA (2008). Daptomycin exerts bactericidal activity without lysis of Staphylococcus aureus. Antimicrob Agents Chemother.

[51] Revest M, Jacqueline C, Boudjemaa R, Caillon J, Le Mabecque V, Breteche A (2016). New in vitro and in vivo models to evaluate antibiotic efficacy in Staphylococcus aureus prosthetic vascular graft infection. J Antimicrob Chemother.

[52] Parra-Ruiz J, Vidaillac C, Rose WE, Rybak MJ (2010). Activities of high-dose daptomycin, vancomycin, and moxifloxacin alone or in combination with clarithromycin or rifampin in a novel in vitro model of Staphylococcus aureus biofilm. Antimicrob Agents Chemother.

[53] Olson ME, Slater SR, Rupp ME, Fey PD (2010). Rifampicin enhances activity of daptomycin and vancomycin against both a polysaccharide intercellular adhesin (PIA)-dependent and -independent Staphylococcus epidermidis biofilm. J Antimicrob Chemother.

[54] Cirioni O, Mocchegiani F, Ghiselli R, Silvestri C, Gabrielli E, Marchionni E (2010). Daptomycin and rifampin alone and in combination prevent vascular graft biofilm formation and emergence of antibiotic resistance in a subcutaneous rat pouch model of staphylococcal infection. Eur J Vasc Endovasc Surg.

[55] Nadrah K, Strle F. Antibiotic combinations with daptomycin for treatment of Staphylococcus aureus infections. Chemother Res Pract. 2011:619321. 10.1155/2011/619321 PMC3265245 22312555

[56] Salem AH, Elkhatib WF, Noreddin AM (2011). Pharmacodynamic assessment of vancomycin–rifampicin combination against methicillin resistant Staphylococcus aureus biofilm: a parametric response surface analysis. J Pharm Pharmacol.

[57] Boudjemaa R, Briandet R, Revest M, Jacqueline C, Caillon J, Fontaine-Aupart MP (2011). New insight into Daptomycin bioavailability and localization in Staphylococcus aureus biofilms by dynamic fluorescence imaging. Antimicrob Agents Chemother.

[58] Khasawneh FA, Ashcraft DS, Pankey GA. In vitro testing of daptomycin plus rifampin against methicillin-resistant Staphylococcus aureus resistant to rifampin. Saudi Med J. 29:1726–9. 19082221

[59] Pogliano J, Pogliano N, Silverman JA (2008). 2012. Daptomycin-mediated reorganization of membrane architecture causes mislocalization of essential cell division proteins. J Bacteriol.

[60] Smith JR, Claeys KC, Barber KE, Rybak MJ (2014). High-dose daptomycin therapy for staphylococcal endocarditis and when to apply it. Curr Infect Dis Rep.

[61] Singh R, Ray P, Das A, Sharma M (2010). Penetration of antibiotics through Staphylococcus aureus and Staphylococcus epidermidis biofilms. J Antimicrob Chemother.

[62] Siala W, Mingeot-Leclercq MP, Tulkens PM, Hallin M, Denis O, Van Bambeke F (2014). Comparison of the antibiotic activities of Daptomycin, vancomycin, and the investigational fluoroquinolone Delafloxacin against biofilms from Staphylococcus aureus clinical isolates. Antimicrob Agents Chemother.

[63] Fowler VG, Miro JM, Hoen B, Cabell CH, Abrutyn E, Rubinstein E (2005). Staphylococcus aureus endocarditis: a consequence of medical progress. JAMA.

[64] Abdelhady W, Bayer AS, Seidl K, Nast CC, Kiedrowski MR, Horswill AR (2013). Reduced vancomycin susceptibility in an in vitro catheter-related biofilm model correlates with poor therapeutic outcomes in experimental endocarditis due to methicillin-resistant Staphylococcus aureus. Antimicrob Agents Chemother.

[65] Huang JH, Hsu RB (2008). Treatment of infective endocarditis caused by methicillin-resistant Staphylococcus aureus: teicoplanin versus vancomycin in a retrospective study. Scand J Infect Dis.

[66] Li N, Zhu L, Xu G, Ge T, Qi F, Li M (2017). Optimal teicoplanin dosage regimens for methicillin-resistant Staphylococcus aureus infections in endocarditis patients and renal failure patients. J Chemother.

[67] Saby L, Laas O, Habib G, Cammilleri S, Mancini J, Tessonnier L (2013). Positron emission tomography/computed tomography for diagnosis of prosthetic valve endocarditis: increased valvular 18F-fluorodeoxyglucose uptake as a novel major criterion. J Am Coll Cardiol.

[68] Fournier PE, Gouriet F, Casalta JP, Lepidi H, Chaudet H, Thuny F (2017). Blood culture-negative endocarditis: improving the diagnostic yield using new diagnostic tools. Medicine (Baltimore).

[69] Fida M, Dylla BL, Sohail MR, Pritt BS, Schuetz AN, Patel R (2019). Role of prolonged blood culture incubation in infective endocarditis diagnosis. Eur J Clin Microbiol Infect Dis.

[70] Rohacek M, Erne P, Kobza R, Pfyffer GE, Frei R, Weisser M (2015). Infection of cardiovascular implantable electronic devices: detection with sonication, swab cultures, and blood cultures. Pacing Clin Electrophysiol.

[71] Oberbach A, Schlichting N, Feder S, Lehmann S, Kullnick Y, Buschmann T, et al. New insights into valve-related intramural and intracellular bacterial diversity in infective endocarditis. PLoS One. 2017;12. 10.1371/journal.pone.0175569 PMC5391965 28410379

[72] Inacio RC, Klautau GB, Murça MA, da Silva CB, Nigro S, Rivetti LA (2015). Microbial diagnosis of infection and colonization of cardiac implantable electronic devices by use of sonication. Int J Infect Dis.

[73] Parsek MR, Singh PK (2003). Bacterial biofilms: an emerging link to disease pathogenesis. Annu Rev Microbiol.

[74] Di Domenico EG, Cavallo I, Pontone M, Toma L, Ensoli F (2017). Biofilm producing Salmonella typhi: chronic colonization and development of gallbladder cancer. Int J Mol Sci.

[75] Di Domenico EG, Toma L, Prignano G, Pelagalli L, Police A, Cavallotti C (2015). Misidentification of streptococcus uberis as a human pathogen: a case report and literature review. Int J Infect Dis.

[76] Di Domenico EG, Farulla I, Prignano G, Gallo MT, Vespaziani M, Cavallo I (2017). Biofilm is a major virulence determinant in bacterial colonization of chronic skin ulcers independently from the multidrug resistant phenotype. Int J Mol Sci.

[77] Rimoldi SG, De Vecchi E, Pagani C, Zambelli A, Di Gregorio A, Bosisio E (2016). Use of dithiothreitol to dislodge bacteria from the biofilm on an aortic valve in the operating theatre: a case of infective endocarditis caused by Staphylococcus aureus and Proteus mirabilis. Ann Thorac Surg.

[78] Oliva A, Nguyen BL, Mascellino MT, D'Abramo A, Iannetta M, Ciccaglioni A (2013). Sonication of explanted cardiac implants device infections improves microbial detection in cardiac devices infections. J Clin Microbiol.

