# Supplementary material for: Microbial biofilm correlates with an increased antibiotic tolerance and poor therapeutic outcome in infective endocarditis
Source: BMC Microbiol. 2019 Oct 21;19:228. doi: 10.1186/s12866-019-1596-2 (PMC6802308; doi:10.1186/s12866-019-1596-2)
Supplement: Supplementary file 1 — Additional file 1:. List and concentration range (μg/ml) of the antibiotics tested. (PPTX 43 kb) [file 12866_2019_1596_MOESM1_ESM.pptx]

## Slide 1
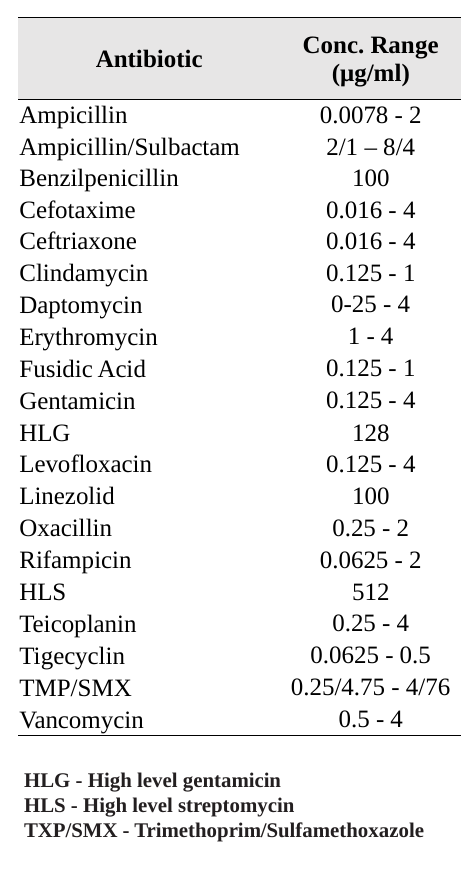

| Antibiotic | Conc. Range (μg/ml) |
| --- | --- |
| Ampicillin | 0.0078 - 2 |
| Ampicillin/Sulbactam | 2/1 – 8/4 |
| Benzilpenicillin | 100 |
| Cefotaxime | 0.016 - 4 |
| Ceftriaxone | 0.016 - 4 |
| Clindamycin | 0.125 - 1 |
| Daptomycin | 0-25 - 4 |
| Erythromycin | 1 - 4 |
| Fusidic Acid | 0.125 - 1 |
| Gentamicin | 0.125 - 4 |
| HLG | 128 |
| Levofloxacin | 0.125 - 4 |
| Linezolid | 100 |
| Oxacillin | 0.25 - 2 |
| Rifampicin | 0.0625 - 2 |
| HLS | 512 |
| Teicoplanin | 0.25 - 4 |
| Tigecyclin | 0.0625 - 0.5 |
| TMP/SMX | 0.25/4.75 - 4/76 |
| Vancomycin | 0.5 - 4 |
HLG - High level gentamicin
HLS - High level streptomycin
TXP/SMX - Trimethoprim/Sulfamethoxazole
