# Supplementary figures and images for: Microbial biofilm correlates with an increased antibiotic tolerance and poor therapeutic outcome in infective endocarditis
Source: BMC Microbiol. 2019 Oct 21;19:228. doi: 10.1186/s12866-019-1596-2 (PMC6802308; doi:10.1186/s12866-019-1596-2)

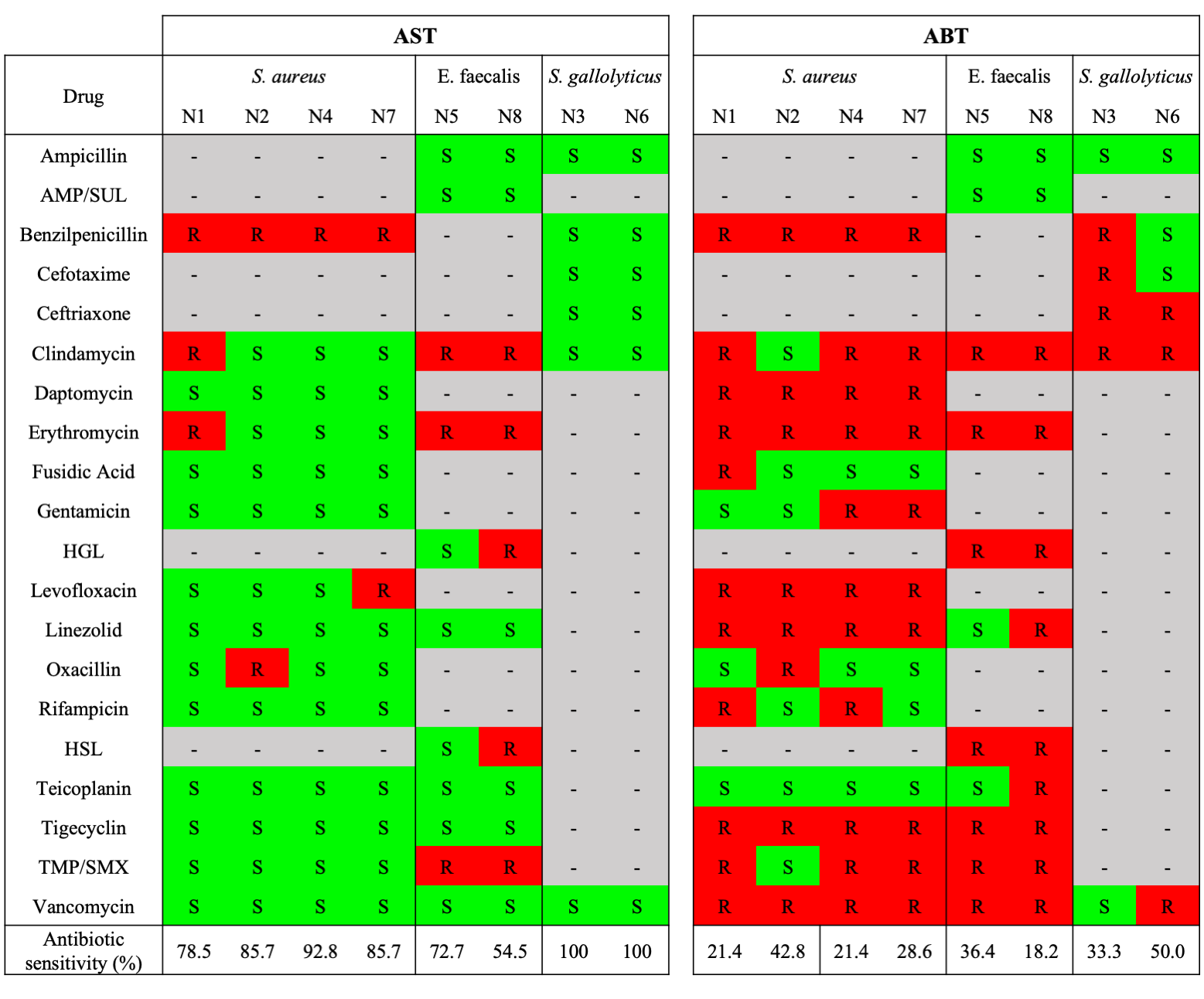

Supplement: Supplementary file 2 — Additional file 2: Comparison between the Antimicrobial susceptibility test (AST) and the Anti-Biofilm Test (ABT). Susceptibility (S) and Resistance profiles of S. aureus (N1, N2, N4 and N7), E. faecalis (N5 and N8) and S. gallolyticus (N3 and N6) clinical isolates to different antimicrobials. Classification was performed according to the European Committee on Antimicrobial Susceptibility Testing clinical breakpoint tables (EUCAST clinical breakpoint table v 9.0). Ampicillin/Sulbactam (AMP/SUL), HLG - High level gentamicin, HLS - High level streptomycin, TXP/SMX - Trimethoprim/Sulfamethoxazole. (TIFF 5897 kb) [file 12866_2019_1596_MOESM2_ESM.tiff]
